# Continuing Effectiveness of Serogroup A Meningococcal Conjugate Vaccine, Chad, 2013

**DOI:** 10.3201/eid2101.140256

**Published:** 2015-01

**Authors:** Kadidja Gamougam, Doumagoum M. Daugla, Jacques Toralta, Cyriaque Ngadoua, Florence Fermon, Anne-Laure Page, Mamoudou H. Djingarey, Dominique A. Caugant, Olivier Manigart, Caroline L. Trotter, James M. Stuart, Brian M. Greenwood

**Affiliations:** Hôpital Général de Référence Nationale, N’Djamena, Chad (K. Gamougam);; Centre de Support en Santé International, N’Djamena (D.M. Daugla, J. Toralta);; Ministere de la Santé Publique, N’Djamena (C. Ngadoua);; Epicentre– Médecins sans Frontières, Paris, France (F. Fermon, A.-L. Page);; World Health Organization Intercountry Support Team, Ouagadougou, Burkina Faso (M.H. Djingarey);; Norwegian Institute for Public Health, Oslo, Norway (D.A.Caugant);; London School of Hygiene & Tropical Medicine, London, UK (O. Manigart, J.M. Stuart, B.M. Greenwood);; University of Cambridge, Cambridge, UK (C.L.Trotter)

**Keywords:** meningitis, meningococcal, serogroup A, vaccine, Africa, bacteria

## Abstract

In 2011, vaccination with a serogroup A meningococcal polysaccharide conjugate vaccine was implemented in 3 of 23 regions in Chad. Cases of meningitis declined dramatically in vaccinated areas, but an epidemic continued in the rest of Chad. In 2012, the remaining Chad population was vaccinated, and the epidemic was halted.

For >100 years, countries in the meningitis belt of Africa have experienced intermittent epidemics of meningococcal meningitis, caused mainly by the serogroup A meningococcus (*1*). After development and prequalification of a new serogroup A meningococcal polysaccharide/tetanus toxoid conjugate vaccine (PsA-TT) in 2009 (*2*), vaccination with PsA-TT across the meningitis belt commenced in 2010, starting with persons 1–29 years of age in Burkina Faso and parts of Mali and Niger (*3*). Little transmission of the serogroup A meningococcus was occurring in these countries at the time of vaccine introduction, making evaluation of its effectiveness difficult. 

In contrast, in Chad, PsA-TT was introduced in the middle of a serogroup A meningococcal epidemic, and vaccination with PsA-TT commenced at the end of 2011, shortly before the 2012 epidemic season. At this time, vaccination of persons 1–29 years of age (target 1.8 million) was undertaken in the capital N’Djamena, Mayo Kebbi Est, and Chari Baguirmi (*4*), designated here as the N’Djamena regions (Figure 1). In 2012, the vaccination program was extended to the rest of the country (target 5.9 million) (Figure 1). During the 2012 meningitis season, the incidence of meningitis decreased by >90% in vaccinated areas compared with the rest of the country, and a similar reduction in the incidence of carriage of serogroup A *Neisseria meningitidis* was found, as reported previously (*4*). We report on the incidence of meningitis during the 2013 meningitis season after vaccination of persons 1–29 years of age in areas with no prior vaccination program. 

**Figure 1:**
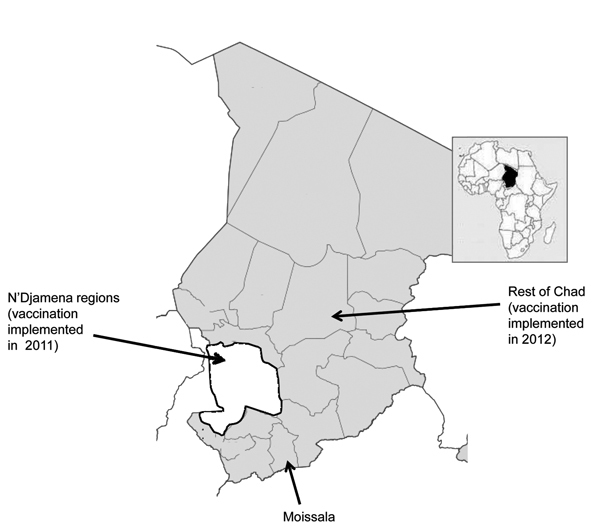
Areas of Chad in which vaccination with serogroup A meningococcal polysaccharide/tetanus toxoid conjugate vaccine was implemented in 2011 (white) and 2012 (gray).

## The Study

In Chad, health districts provide aggregated weekly data on meningitis and other notifiable diseases to the Ministry of Health. This system was reinforced in 2012 and 2013 by case-based surveillance supported by the Centre du Support en Santé Internationale in the N’Djamena regions, and also by Médecins sans Frontières in Moissala, a district ≈800 km from N’Djamena (Figure 1). Cerebrospinal fluid (CSF) specimens obtained from persons with suspected cases of meningitis were transported to the national reference laboratory in N’Djamena. Isolated strains of *N. meningitidis* were sent to the World Health Organization (WHO) Intercountry Support Team, Ouagadougou, Burkina Faso, and to the WHO Collaborating Centre for Reference and Research on Meningococci in Oslo. Information about the laboratory methods used to isolate and characterize meningococci is provided elsewhere (*4*). Data from the 2009 census were used to calculate incidence rates. We used a negative binomial regression model to assess the effect of PsA-TT on the incidence of meningitis in the N’Djamena regions in 2012 and in the whole country in 2013; we used weekly data obtained during the epidemic period (weeks 1–26) during 2009–2013.

**Figure 2:**
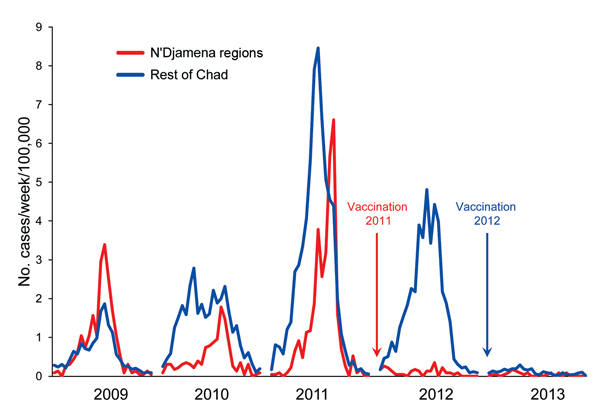
Incidence (no. cases/100,000 population) during weeks 1–26 of reported cases of meningitis in regions of Chad where persons 1–29 years of age were vaccinated with serogroup A meningococcal polysaccharide/tetanus toxoid conjugate vaccine at the end of 2011 and in 2012.

The incidence of meningitis in Chad during 2009–2013 and its association with the introduction of PsA-TT are shown in Figure 2. During weeks 1–26 of 2012, the incidence of reported meningitis among persons in all age groups in the N’Djamena regions that received vaccine was 2.5 cases/100,000 population (57/2.3 million); during the previous year, incidence was 31.8/100,000 (732/2.3 million). Meningitis incidence remained low in the N’Djamena regions in 2013 at 1.1/100,000 (25/2.3 million). In the rest of the country, in which vaccination was implemented during 2012 only, meningitis incidence decreased from 43.8/100,000 (3,809/8.7 million) in weeks 1–26 of 2012 to 2.8/100,000 (247/8.7 million) during the same period in 2013, a 96% reduction (p<0.0001). The incidence rate ratio for vaccinated versus unvaccinated populations was estimated by using data across the whole study period with a negative binomial regression model; the incidence rate ratio was 0.104 (95% CI 0.052–0.207).

Fewer CSF specimens were submitted to the national reference laboratory in N’Djamena in 2013 than in 2012 (Table), but the proportion of reported cases for which CSF samples were submitted increased from 8.3% (273/3,308) in 2010, 7.9% (516/6,540) in 2011, and 9.5% (366/3,866) in 2012, to 39.3% (106/272) in 2013 (Pearson χ^2^ p<0.0001). The proportion of reported cases for which CSF samples were submitted from the N’Djamena regions was highest in 2010, when the main pediatric unit for N’Djamena was in the same hospital as the national reference laboratory (the unit moved to another hospital in 2011) and in 2012, when case-based surveillance was introduced. In Moissala, the proportion of cases for which CSF samples were submitted increased from 22% (74/341) in 2012 to 119% (56/47) in 2013, the latter figure being attributed to undernotification. During weeks 1–26 of 2013, a total of 106 CSF specimens were received by the national reference laboratory; 13 yielded *Streptococcus pneumoniae*, 4 *Haemophilus influenzae* type b, 2 *N. meningitidis* serogroup W, and 1 (obtained from a 3-year-old child who had not received PsA-TT) serogroup A *N. meningitidis*. Four infections were caused by other pathogens. This finding differed markedly from those of previous years (2010−2012), when the predominant organism was *N. meningitidis* serogroup A, and only a few cases caused by *N. meningitidis* serogroup W and *S. pneumoniae* were also identified. The predominance of serogroup A infection in Chad during 2010–2012 was confirmed among the CSF specimens examined at the National Institute of Public Health in Oslo. All fully characterized serogroup A strains were porA 20.9, FetA F3.1, sequence type (ST) 7 (ST5 complex), and all serogroup W strains were porA 5.2, FetA F1–1, ST11 (ST11 complex). Although national reference laboratory data were not available in Chad for 2009, the predominant organism identified from CSF specimens received at the National Institute of Public Health in Oslo in 2009 was *N. meningitidis* serogroup W (11/14 serogrouped strains), also porA 5.2, FetA F1–1, and ST11 (ST11 complex) (*5*).

**Table:** Diagnoses of suspected meningitis cases from CSF specimens, Chad, weeks 1–26, 2010–2013*

Location, year	No. reports of suspected meningitis	CSF specimens, no. (%)	Diagnosis†						
			*N. meningitidis*				*S. pneumoniae*	*H. influenzae* type b	Other
			A	W	X	Other			
N'Djamena regions									
2010	268	158 (58.9)	27	2	0	2	8	0	0
2011	732	163 (22.3)	45	1	1	0	6	0	0
2012	57	37 (64.9)	0	0	2	0	0	0	0
2013	25	7 (28.0)	0	0	0	0	1	0	0
Rest of Chad									
2010	3,040	65 (2.1)	28	1	0	0	2	0	0
2011	5,808	353 (6.1)	110	1	1	0	0	1	0
2012	3,809	329 (8.6)	59	4	0	0	4	0	0
2013	247	99 (40.1)	1	2	0	0	12	4	4‡

*Diagnoses were based on cerebrospinal fluid (CSF) specimens received at the national reference laboratory in N’Djamena, Chad. Vaccination with serogroup A meningococcal polysaccharide/tetanus toxoid conjugate vaccine was implemented in the N’Djamena regions in 2011 and in all other regions of Chad in 2012. No reference laboratory data are available for 2009. *N., Neisseria; S., Streptococcus; H., Haemophilus.* †Based on culture or latex agglutination. ‡*Acinetobacter baumanii*, *Salmonella paratyphi* A, *Staphylococcus hominis*, *Staphylococcus aureus.*

## Conclusions

We previously reported a >90% reduction in incidence of meningitis among vaccinated populations in Chad in 2011 (*4*). Here we report a similar reduction in the incidence of meningitis in 2013 from that in 2012 for populations vaccinated only during the second year of the vaccination campaign. The epidemic curve (Figure 2) suggests that by 2013, the *N. meningitidis* serogroup A epidemic in Chad was waning and that fewer cases of serogroup A meningitis would have occurred during 2013 than during 2012, even in the absence of vaccination. However, the incidence of meningitis dropped lower in 2013 than would have been expected as a result of a natural decline, and only 1 serogroup A isolate was obtained at the national reference laboratory despite improved CSF sampling. This finding provides strong additional evidence of vaccine effectiveness for preventing serogroup A meningococcal disease in Chad.

If the effectiveness of PsA-TT vaccination seen in Chad and Burkina Faso (*6*,*7*) is replicated across the meningitis belt of Africa and if vaccine coverage can be sustained through introduction of PsA-TT into the infant immunization program and/or through mass campaigns, serogroup A epidemics could disappear from the meningitis belt. However, past experience has shown that meningococci belonging to serogroups C, W, or X can cause substantial epidemics (*8*–*10*); therefore, continuing surveillance will be needed to determine how the epidemiology of meningococcal disease in the meningitis belt of Africa is changed by the successful introduction of PsA-TT (*11*). The Chad Ministry of Health has approved a plan to support and develop case-based surveillance in the N’Djamena regions, Moissala, and 3 other selected health districts.
